# Mean plant toxicity modulates the effects of plant defense variability

**DOI:** 10.1002/ecy.70012

**Published:** 2025-02-04

**Authors:** Vincent S. Pan, Kadeem J. Gilbert, William C. Wetzel

**Affiliations:** ^1^ Department of Integrative Biology Michigan State University East Lansing Michigan USA; ^2^ W. K. Kellogg Biological Station Michigan State University Easting Lansing Michigan USA; ^3^ Ecology, Evolution, and Behavior Program Michigan State University Easting Lansing Michigan USA; ^4^ Department of Plant Biology Michigan State University East Lansing Michigan USA; ^5^ Land Resources and Environmental Sciences Montana State University Bozeman Montana USA

**Keywords:** *Arabidopsis thaliana*, glucosinolate, herbivory, intraindividual variation, intraspecific trait variation, mean–variance interaction, nonlinear averaging, plant defense, *Trichoplusia ni*, variance decomposition

## Abstract

Plant trait variation is thought to suppress herbivore performance, but experiments typically manipulate only a single mean level of the trait. We manipulated the mean and variation of the concentration of a plant toxin in a model plant–herbivore system across three field and greenhouse experiments. Plants with leaves painted with a higher mean toxin concentration exhibited increased fitness and resistance to herbivores; however, at high mean concentrations, variation reduced the defensive effect, while at lower mean concentrations, variation enhanced it. This reversal aligns with models that include herbivore food selectivity, but our simulations revealed that the benefits of food selectivity for herbivores were minimal. Instead, nonlinear averaging and physiological tracking effects likely drove patterns in plant fitness and resistance to herbivores. We suggest that high defense variation in plants may be a widespread defensive phenotype, but for well‐defended plants, variation may inadvertently promote herbivore niche expansion.

## INTRODUCTION

Chemical defenses, a key plant functional trait that drives interactions with herbivores, are highly variable in nature at all scales (Wetzel et al., 2023). Long‐standing hypotheses propose that plant trait variability may be adaptive as a defense against herbivores (Herrera, 2009; Karban et al., 1997; Schultz, 1983; Shelton, 2004; Wetzel et al., 2023; Whitham, 1981), and emerging evidence suggests that trait variation can suppress individual herbivore performance (Karban et al., 1997; Shelton, 2000; Wetzel et al., 2016) and herbivore population growth rate (Pearse et al., 2018). Exciting new work is increasingly elucidating the heritable genetic basis of trait variance (Ayroles et al., 2015; Herrera et al., 2022; Metcalf & Ayroles, 2020), with a typical evolvability (genetic coefficient of variation) around 30% in a review (Hill & Mulder, 2010), but the plant fitness benefit has yet to be demonstrated. For a trait to be defensive, a plant must benefit from the trait in the presence of herbivores, but resistance to herbivores is a poor predictor of plant defense. For example, low nutritive quality tissue or anti‐digestive compounds can trigger compensatory feeding in herbivores and therefore reduce plant fitness (Augner, 1995).

**FIGURE 1:**
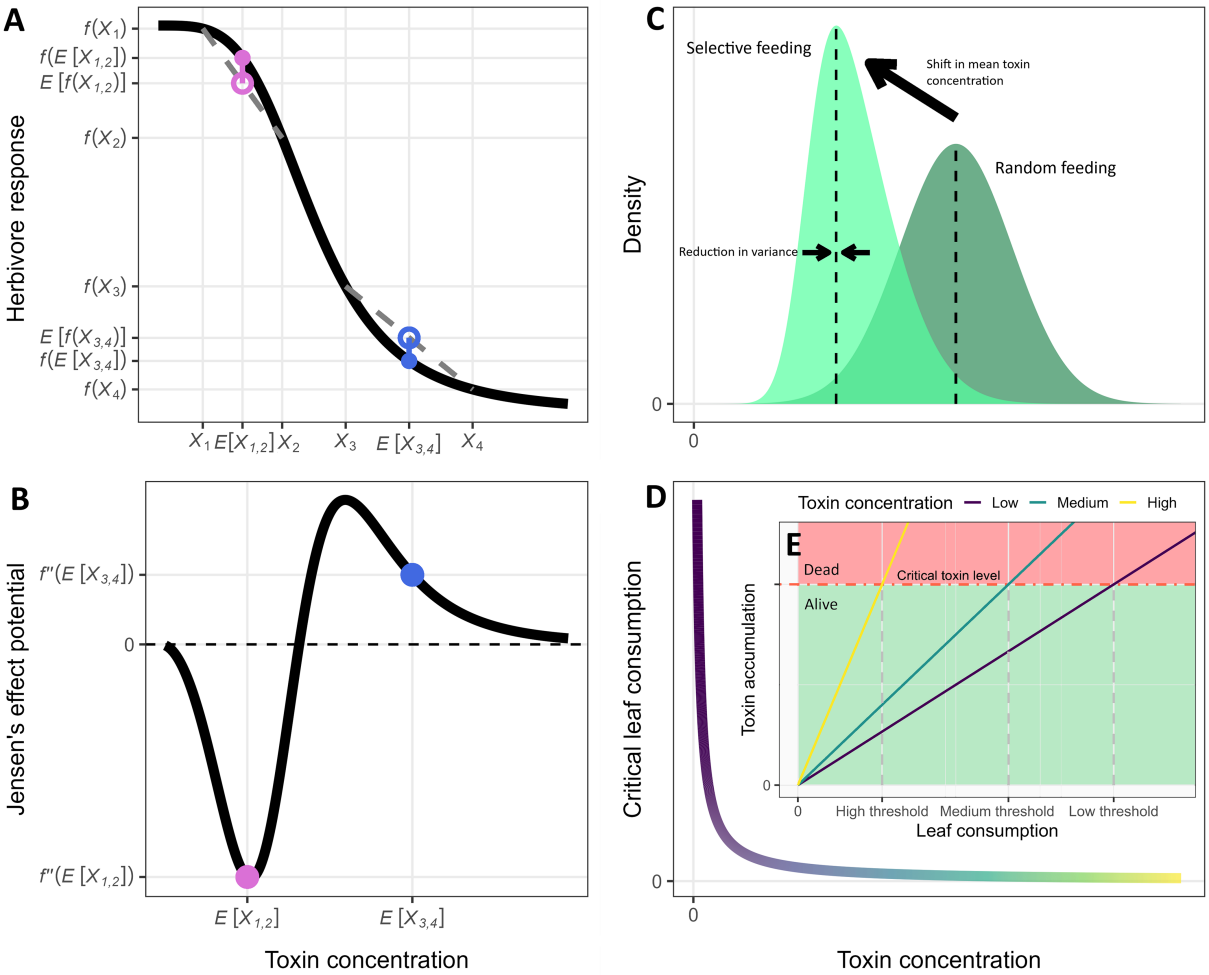
(A) A depiction of Hill's equation (denoted as $f\left(x\right)$) from toxicology where the response function has both concave and convex regions along different toxin concentrations *x*. The response to toxin concentration that varies between two values $X_{1}$ and $X_{2}$ has a lower average (open circle) than the response to the average toxin concentration of the two values (closed circle). The converse is true for the concave region where the toxin concentration varies between $X_{3}$ and $X_{4}$ . Jensen' effects (nonlinear averaging effects) are shown as the distance between the open and closed circles. (B) Jensen's effect potential (second derivative) of the same Hill's equation, showing the degree of local convexity. (C) With herbivore selective feeding, the distribution of toxin concentration experienced by the herbivore changes. The change can be in terms of a reduction in both mean and variance of toxin concentration (Appendix S1: Section S3). (D) The critical leaf consumption threshold, above which herbivores are poisoned, has an inverse relationship with toxin concentration. Thus, a small increase in toxin concentration, when the mean toxin concentration is already high, causes a smaller decrease in critical herbivore leaf consumption than when the mean toxin concentration is lower. (E) This inverse relationship is a direct consequence of the fact that toxin concentration affects the speed at which a feeding herbivore reaches its critical toxin accumulation level (*a la* Wolkovich et al., 2021).

Even if plant defense variation provides a fitness benefit, much remains unclear about the mechanisms. To date, three major mechanisms of the effect of defense variability on herbivore performance have been identified: nonlinear averaging, selective feeding, and constraints to physiological tracking. Various studies have found their proposed mechanism of overriding importance, predicting a range of effects on herbivores (Pearse et al., 2018; Thiel et al., 2021; Wetzel et al., 2016). Nonlinear averaging (also known as Jensen's inequality) occurs when the herbivore performance function at different levels of a plant defense is concave or convex (Figure 1). In the presence of variability, nonlinear averaging of a concave function suppresses herbivore performance, whereas a convex function enhances it (Karban et al., 1997). On the other hand, selective feeding on less defended plant tissues can allow herbivores to circumvent toxins when variation in the concentration of those toxins creates “weak links” of underdefended tissues, thereby enhancing herbivore performance (Thiel et al., 2020, 2021). Finally, constraints on physiological tracking may prevent herbivores from acclimating their physiology to counter different levels of plant defense (Kingsolver & Woods, 2016; Koussoroplis et al., 2017; Pearse et al., 2018). For instance, the detoxification response to dietary toxins in *Trichoplusia ni* has a lag of 12 h (Fang et al., 2007), during which the insect either cannot feed or face toxin levels it cannot optimally cope with.

In the presence of plant defense variability, all three mechanisms likely operate simultaneously. A more nuanced understanding of the effect of defense variability necessitates understanding how the relative contribution of each of these mechanisms, as well as other unknown mechanisms, changes and combines to affect both herbivore and plant fitness in different contexts. These mechanisms can also create complex interactions. For instance, selective feeding may modulate the effect of nonlinear averaging through a reduction in the variation in dietary toxin intake or a shift in the mean of the distribution of dietary toxin to a more nonlinear region of the dose–response function (Figure 1C). A key challenge in empirically assessing such a nuanced, multicausal question is separating the effect of defense variability into its causal components, beyond testing for each component's presence. We lack both the method and appropriate data to do so.

To illustrate how each mechanism changes in importance, and to identify key contexts that alter the effect of trait variability, we consider the effect of variability under different mean defense levels. Our study heeds increasing calls to incorporate variation into ecology (Bolnick et al., 2011; Shoemaker et al., 2020) but also includes considerations for mean–variance interactions and relationships that have received much less attention (Benedetti‐Cecchi, 2003), as studies of plant trait variation typically hold the trait at one level of mean. We predict that defense variation enhances resistance to herbivores and plant fitness when the mean defense level is low but reduces them when the mean defense level is high. Our prediction results from the following hypotheses. First, dose–response functions are often both concave and convex (Figure 1A, Di Veroli et al., 2015), with the function more likely to be concave at lower toxin doses where it is likely close to a fitness maximum. This implies that, depending on the mean plant defense level, variation in the defense trait can either enhance or reduce herbivore performance via nonlinear averaging (Figure 1B). Second, given that herbivore preference is expected to be stronger when plants are more toxic (because the benefit of selectivity outweighs the cost), we might expect that toxin variability enhances herbivore performance when the overall mean level of plant toxicity is higher. Third, the effect of constraints on the herbivore's ability to physiologically track variation in defenses is likely to be weaker at higher defense levels because the effect of a plant toxin concentration at high levels tends to be saturating. The diminishing effect results from the fact that a toxin suppresses herbivore performance through accumulation in the herbivore's system (Figure 1D,E). Thus, the sensitivity to additional “unexpected” defenses (i.e., marginal effect of defenses) would tend to be weaker at high mean toxin concentrations. This phenomenon of reduced sensitivity is analogous to the reduced plant phenological temperature sensitivity at higher temperatures (Wolkovich et al., 2021). Taken together, if true, our hypotheses would suggest that some of the previous conclusions about the benefits of plant variability regarding crop diversification (e.g., Wetzel et al., 2016) and trait evolution may require reinterpretations based on the mean plant quality. For example, increasing plant functional diversity in agricultural systems where pests are already strongly bottom‐up regulated might be counterproductive for pest suppression.

We experimentally tested whether toxin variation enhances plant fitness when the mean toxin concentration is low but reduces plant fitness when the mean concentration is high. Accordingly, we conducted a 2‐month‐long caged field experiment at both the intraindividual and inter‐individual scales, as which scale of variation is ecologically relevant remains an open question and has implications for the unit of selection. Although a group of individuals is not technically a unit of selection, inter‐individual variation is important for frequency‐dependent processes, such as the maintenance of neighborhood‐level phytochemical overdispersion (Wang et al., 2023). We then tested in two greenhouse experiments whether the potential effect on plant fitness arises from the suppression of herbivore performance. Finally, we introduced a method using counterfactual simulation to partition the direct effect of nonlinear averaging, selective feeding, their interaction, and residual effects (including physiological tracking effects). As a proof of concept, we tested these hypotheses using a model system consisting of the plant *Arabidopsis thaliana* (Brassicaceae), its herbivore *T. ni* (Noctuidae, cabbage looper), and its chemical defense sinigrin (a type of glucosinolate). Modeling suggests that glucosinolate production reduces biomass production in *A. thaliana* by 15% (Bekaert et al., 2012), but it is nevertheless maintained and selected for in the presence of herbivores (Mauricio & Rausher, 1997). Moreover, quantitative trait loci that control the stochastic variation independently of the mean in glucosinolate have been identified (Jimenez‐Gomez et al., 2011). Hence, the variation and mean of glucosinolate have different genetic bases for selection. Together, our study reveals an important context dependency (i.e., mean defense levels) in the effects of plant defense variation and provides a means to generalize the understanding of this context dependency.

## METHODS

### Plant fitness in response to defense variability

To test the interactive effect of defense variability and mean defense levels on plant fitness, we conducted a field experiment with wild‐type Col‐1 (Columbia) *A. thaliana* from July 7 to September 1, 2022. We grew 612 *A. thaliana* from June 13 to July 5 in a greenhouse in individual 7.6 × 7.6 cm square pots. We filled the soil to the brim to allow the herbivores to move freely from plant to plant. One week before bolting, we randomly grouped six plants and a second instar *T. ni* caterpillar each in a 5‐gallon mesh bag and applied one of six treatments in a stratified random block fashion (*n* = 102 cages, 15–18 cages/treatment; Appendix S1: Figure S1a). The replicated treatments consisted of a factorial cross between two or three levels of toxin variation (constant‐, inter‐, or intraindividual variation) and two levels of mean cage‐level toxin variation (low or high). The constant treatment kept intra‐ and inter‐individual sinigrin concentration constant at the cage‐level mean (low μ = 0.66, high μ = 1.89 nmol/mg FW^−1^). These concentrations roughly correspond to the concentration of total glucosinolate found in old (L3‐5) and mature (L6‐8) leaves of wild‐type *A. thaliana*, respectively; neither concentration is high relative to the concentrations in young leaf tissues and other *A. thaliana* genotypes (Hunziker et al., 2021; Panthee et al., 2011). The inter‐individual variation treatment kept the intraindividual sinigrin concentration constant, but each plant either received a high or low (μ ± 0.61 nmol/mg) dose. The intraindividual variation treatment kept inter‐individual plant sinigrin concentration the same as the cage mean, but each leaf received either a high or low (μ ± 0.61 nmol/mg) dose.

We deployed the sinigrin (sinigrin potassium salt or sinigrin hydrate) by mixing the crystals in deionized water and painting the solution on the adaxial surface of the leaves (Appendix S1: Section S1, Appendix S1: Table S1). This design has the advantage of being a direct experimental manipulation of the defense level, whereas previous studies have manipulated defenses indirectly using genotypes of mutants with different defense levels, which may have been correlated with other traits via pleiotropy (Hunziker et al., 2021; Schuman et al., 2015). Foliar application of glucosinolates is also unlikely to cause phytotoxic effects independent of herbivory because glucosinolates only become biologically active when they react with myrosinase released in ruptured leaf tissue (Chew, 1988). When plants were 3 weeks old, we moved them into cages in the field at MSU Kellogg Biological Station (42.410, −85.391), burying the bags and plots flush with the soil surface. At the end of the experiment, we counted the number of second‐generation *A. thaliana* in each pot. We used this count as a measure of absolute fitness of each plant as most seeds land directly below their mother plant.

### Herbivore performance in response to defense variability

To assess the interactive effect of defense variability and mean defense on herbivore performance, we performed a similar experiment in a greenhouse. Having demonstrated the treatment effects under realistic field conditions, this follow‐up experiment offers more detailed mechanistic insights under a more controlled environment. We conducted the experiment in two sessions (May 9–10, 2023, May 16–17, 2023) using 3‐week‐old *A. thaliana* and third or fourth instar *T. ni* (*n* = 412 cages, 103 cages/treatment). We applied the same treatments as before but with only two levels of mean toxin treatment (low or high) crossed with two levels of variation treatment (constant or within‐plant variation) and only one plant per cage (Appendix S1: Figure S1b). For each plant, we used a piece of wire to mark the first high dose leaf and painted every other leaf with a high dose. We packed the cages densely in trays of 30 plants, such that the shade from the cages essentially halted leaf expansion and production during our 2‐day experiment. We measured the number of leaves on each plant and the weight of each caterpillar before the experiment. After allowing the caterpillar to feed for 24 h, we reweighed each caterpillar and calculated hourly relative growth rates (RGR). We then allowed caterpillars to feed ad libitum on artificial diets in individual containers after the experimental period and recorded pupation time. We also visually scored the proportion herbivory on each leaf. Because we had marked the order of leaf painting, we were able to deduce which leaf corresponded to which dose treatment we applied.

### Characterization of dose response function

To estimate the effect of nonlinear averaging, we characterized herbivore performance and herbivory as a function of different doses of applied sinigrin. In a separate greenhouse experiment, we caged individual *A. thaliana* with a single third or fourth instar *T. ni* for 36 h. We applied 10 different dose levels of sinigrin (0.031–16.1 nmol/mg) on the leaves of 60 plants (*n =* 60 cages, 6 cages/dose level) (Appendix S1: Figure S1c). We measured the same variables as in our previous greenhouse experiment.

### Statistical analyses

To determine whether glucosinolate variability affected plant fitness, herbivory, and herbivore resistance, and whether these effects depended on the mean cage‐level dose treatment, we fitted measures of plant and herbivore performance and herbivory against mean cage dose treatment, variation treatment, and their interaction using Bayesian hierarchical generalized additive models (package *brms*; Bürkner, 2017). We used weakly regularizing priors for all estimated parameters. We used a log‐linked negative binomial distribution for *A. thaliana* recruitment, identity‐linked Gaussian distributions for caterpillar RGR and logit‐transformed proportion herbivory, and a log‐linked gamma distribution for pupation rate (1/τ, where τ is days to pupation). In all models, we included log transformed pre‐experiment caterpillar weight as a smoothed covariate using thin‐plate splines (Wood, 2003) as we detected significant nonlinearity in exploratory analyses. For the analysis of RGR, pupation rate, and herbivory, we also included log number of pre‐experimental leaves and experimental session as a covariate. We allowed the effect of caterpillar weight to vary between sessions, which significantly improved fit. For the analysis of recruitment, experimental block and cage nested within block were added as random intercepts.

To determine whether herbivores exhibit greater food selection at higher mean cage dose levels, we examined the relative amount of herbivory on leaves painted with a high or low dose in the intraindividual variation treatment (*n* = 1475 leaves, 206 cages). We fitted logit‐transformed individual leaf herbivory against mean cage dose treatment, leaf level dose treatment, their interaction, and other covariates detailed above as fixed effects. Individual plants were added as a random intercept.

To estimate dose–response functions, we fitted RGR, pupation rate, and herbivory in generalized additive models as before. We added the number of leaves as a fixed effect and caterpillar pre‐weight and log sinigrin dose as smoothed terms. We imposed a maximum of four basis dimensions on dose to prevent overfitting, as in Wetzel et al. (2016).

### Variation effect decomposition

We performed counterfactual simulations on responses that were significantly affected by the level of applied sinigrin to partition the relative effects of different variability mechanisms outlined in the introduction. Our approach accords similar efforts in other fields of ecology, such as coexistence theory and thermal ecology, concerned with the investigation of fluctuation dependent mechanisms (Ellner et al., 2016, 2019; Koussoroplis et al., 2017). To perform these simulations, we derived the following equations for the marginal effects of nonlinear averaging ($J_{r}$), selective feeding (S), and the interaction between nonlinear averaging and selective feeding ($J_{S}$) (see also Appendix S1: Section S2 for more details).

Let $f\left(x\right)$ be some dose–response function of the toxin concentration *x*. We define $X_{o}$ as the observed distribution of dietary toxin dose levels experienced by an herbivore (with selective feeding) and $X_{r}$ as the distribution of dietary toxin dose levels where selective feeding does not occur. By definition, the main effect of nonlinear averaging (independent of selective feeding) is equal to $J_{r}≔E\left[f\left(X_{r}\right)\right]-f\left(E\left[X_{r}\right]\right),$ where $E\left[\cdot \right]$ denotes the mean. In the presence of selective feeding, the effect of nonlinear averaging is also by definition $E\left[f\left(X_{o}\right)\right]-f\left(E\left[X_{o}\right]\right)$, making the marginal effect of the interaction between selective feeding and nonlinear averaging, $J_{S}=E\left[f\left(X_{o}\right)\right]-f\left(E\left[X_{o}\right]\right)-J_{r}.$ The main effect of selective feeding, independent of nonlinear averaging, is then, $S=f\left(E\left[X_{o}\right]\right)-f\left(E\left[X_{r}\right]\right).$ Interpreted biologically, it is the effect on herbivore response due to ingesting a reduced mean level of toxin. Adding up these predicted effects, we get, $\hat{T}≔J_{r}+S+J_{S}.$ Comparing this predicted total effect to the actual observed total effect of variability $T_{o}$ allowed us to estimate the effects of the omitted mechanisms (which include constraints to physiological tracking, among others) ϵ, $T_{o}≔\hat{T}+\epsilon .$

For each response variable at each mean dose treatment level, we estimated $f\left(x\right)$ using generalized additive models we fitted to data from the dose response experiment. We incorporated the uncertainty in $f\left(x\right)$ by using 500 posterior draws in the simulation. We estimated $P\left(X_{o}\right)$ using the treatment doses weighed by the estimated herbivore preference (mean proportion herbivory) for each within‐plant toxin dose from our herbivore preference model. For $P\left(X_{r}\right)$, we set the relative preference to zero and used mean herbivory as frequency weights for the two within‐plant toxin doses. To incorporate our uncertainty in the preference estimate, for each posterior draw of $f\left(x\right)$, we repeated the simulation using 500 posterior draws from the preference model. We used the above equations to partition the marginal effect of variability of each mechanism ($J_{r},S,J_{S}$) for each mean dose treatment level. We summarized all simulations by examining the mean and 89% credible intervals (CI) of the 250,000 simulations. All analyses were performed in R version 4.3.1 (R Core Team, 2023).

## RESULTS

### Plant fitness response to defense variability

Plant individuals in cages that received a greater mean cage‐level concentration of sinigrin had higher fitness, with a greater number of offspring in the second generation (mean [89% CI]; percent change = 120% [20%, 350%], Bayes factor [BF] = 50), confirming that applied sinigrin acted as a plant defense.

**FIGURE 2:**
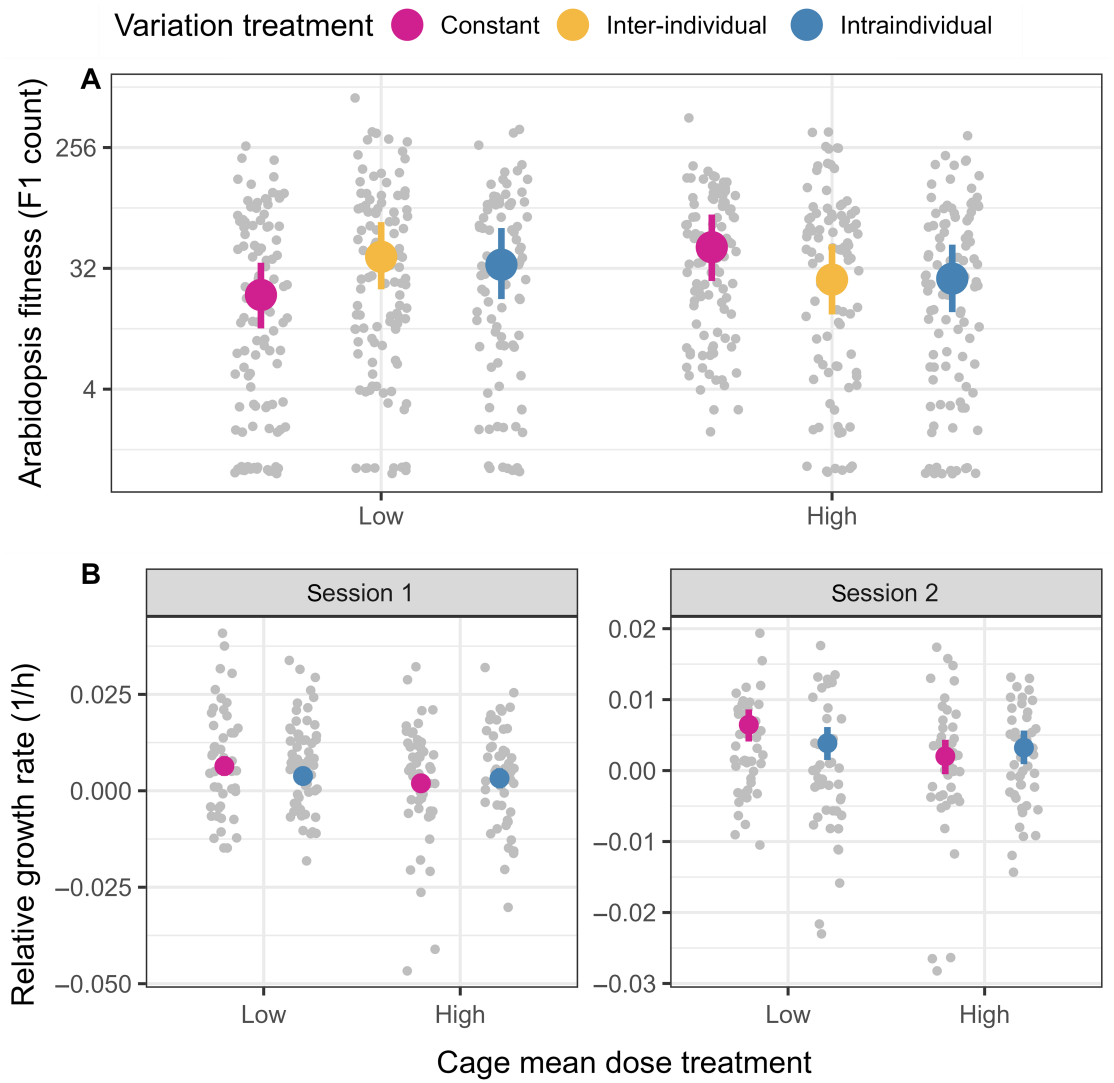
Exogenous sinigrin variation treatments increased plant fitness (A) and decreased herbivore relative growth rate (B) at low mean cage‐level sinigrin dose, but decreased plant fitness and increased herbivore relative growth rate at high mean cage‐level sinigrin dose. Colored points represent mean estimates. Colored bars represent 89% credible intervals. Each gray point represents a plant or a caterpillar.

As we hypothesized, the mean and variation of exogenous sinigrin concentrations influenced *A. thaliana* fitness interactively (Figure 2A) at both inter‐individual (BF = 44) and intraindividual scales (BF = 25). At low mean cage‐level concentrations, inter‐individual variation increased recruitment by 94% ([−4.0%, 290%], BF = 14) and intraindividual variation increased recruitment by 70% ([−18%, 250%], BF = 6.9) relative to the constant treatment. At high mean cage‐level concentrations, however, inter‐individual variation reduced recruitment by 43% ([−73%, 20%], BF = 7.8) and intraindividual variation reduced recruitment by 41% ([−71%, 20%], BF = 8.5) relative to the constant treatment.

### Herbivore response to defense variability

In our greenhouse experiment, caterpillars that fed upon plants painted with a greater mean sinigrin concentration had lower RGR (standardized mean difference = −0.38 standard deviation (SD), [−0.56 SD, −0.20 SD], BF = 3999), but not a lower pupation rate (percent change = 0.49% [−0.02%, 3.0%], BF = 1.6). Moreover, plants treated with more sinigrin had lower odds of herbivory (−71% [−45%, −85%], BF = 666). These results confirmed that applied sinigrin acted as an herbivore resistance trait.

Similar to plant fitness, mean and variation in sinigrin concentration affected *T. ni* performance, specifically RGR, interactively (Figure 2B). We found strong evidence that mean sinigrin concentration altered the effect of intraindividual sinigrin variation on caterpillar RGR (BF = 51), but not the effect on pupation rate (BF = 0.42) or plant herbivory loads (BF = 1.0). At low mean sinigrin concentrations, variation reduced *T. ni* RGR over 24 h by −0.22 SD ([−0.40 SD, −0.042 SD], BF = 42), but increased it by 0.11 SD ([−0.073 SD, 0.29 SD], BF = 4.9) when the mean concentration was high. There was no evidence that variation affected pupation rate or herbivory at low (pupation: 0.76 [−0.020%, 3.5%], BF = 0.49; herbivory: 3.4% [−46%, 99%], BF = 0.87) or high mean sinigrin concentrations (pupation: 0.0% [−0.030%, 2.2%], BF = 0.63; herbivory: 3.7% [−45%, 93%], BF = 1.13).

When plant defense was variable among leaves within a plant, herbivores were able to circumvent plant defenses by feeding selectively on leaves painted with a lower sinigrin concentration. However, this food preference was stronger when the mean toxin concentration was higher (90% [5.6%, 250%], BF = 23; Appendix S1: Figure S2). In the high cage‐level mean treatment, herbivores preferred leaves painted with a lower dose of sinigrin 106% more than leaves painted with a higher dose of sinigrin ([35%, 220%], BF = 307). In contrast, there was no evidence that herbivores preferred leaves painted with a lower dose of sinigrin in the low cage‐level mean concentration treatment (7.5% [−31%, 65%], BF = 1.52).

### Partitioning mechanisms of defense variability effects

**FIGURE 3:**
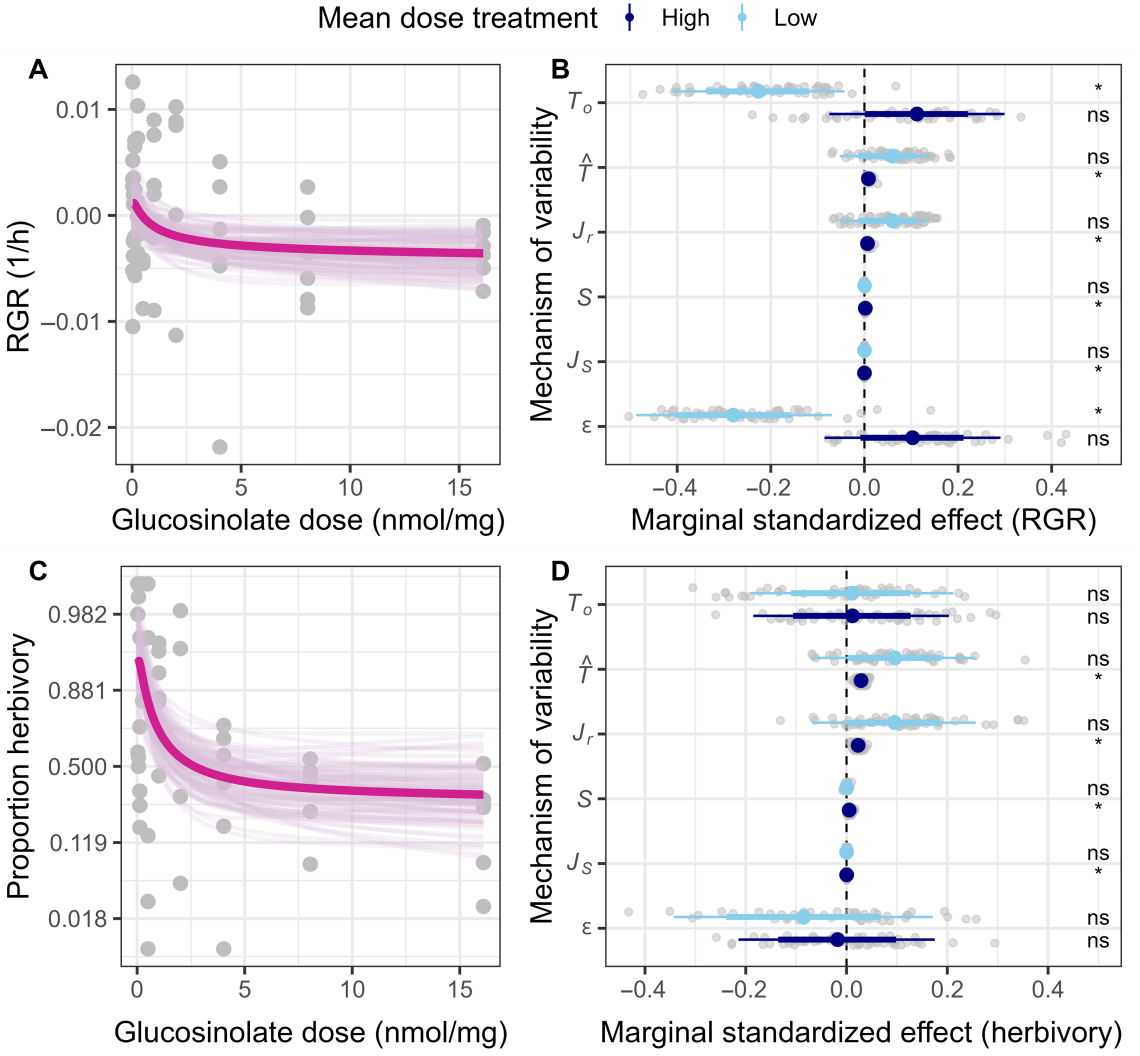
(A, C) Herbivore response functions to exogenously applied sinigrin. Dark thick lines and light thin lines indicate mean estimates and 100 random posterior samples. Each point is a cage. The spacing of the axes was transformed to the scale at which the marginal effects of variability are interpreted. (B, D) Results of counterfactual simulations, partitioning the marginal effect of variability treatment via Equations (2)–(6). Colored points, thin bars, and thick bars represent 50%, 66%, and 89% CIs, respectively. A random sample of 100 simulations are plotted as gray points. An asterisk indicates whether the 89% CIs do not overlap zero at the black dashed line. “ns” is used to denote an otherwise nonsignificant effect. We standardized the effects with the standard deviation of the link transformed response variable. $T_{o}$, $\hat{T}$, and ϵ correspond to observed and predicted total effects and residual effects, respectively. $J_{r}$, S, and $J_{S}$ correspond to the three mechanisms that comprise the predicted total effect, pure nonlinear averaging, selective feeding, and interaction between nonlinear averaging and selective feeding, respectively.

Effects of defense variability on herbivore performance may be due to nonlinear averaging, selective feeding, their interactions, and unmeasured effects, which include physiological effects. To partition these mechanisms, we performed counterfactual simulations using significant dose–response functions for RGR (BF = 7.4; Figure 3A) and herbivory (BF = 349; Figure 3C), but not pupation rate (BF = 0.54). The total effect of variability $\hat{T}$ through nonlinear averaging, food selectivity, and their interaction in the high mean dose treatment group were significantly positive. However, to our surprise, their effect sizes nevertheless were quite weak and not likely to be biologically meaningful (Figure 3B,D). For herbivore RGR in the high mean treatment, the predicted total effect was much smaller in magnitude than the observed total effect of variability ($T_{o}$), although this difference was not distinguishable from zero (Figure 3B; 0.10 SD [−0.085 SD, 0.29 SD]). Conversely, in the low mean treatment, we detected significant total missing effects unaccounted for (ϵ) that corresponds to a −0.28 SD reduction ([−0.49 SD, −0.070 SD]) in RGR over 24 h. This result is consistent with the negative effect of constraints on physiological tracking.

Across both high and low mean treatment groups and both responses, we found that the main effect of nonlinear averaging ($J_{r}$) was positive and was the dominant effect among the three explicit mechanisms of variability that we partitioned (Figure 3B,D). The main effect of nonlinear averaging significantly increased caterpillar RGR by a trivial 0.0071 SD ([0.0020 SD, 0.014 SD]) and the odds of herbivory by 7.8% ([4.1%, 11%]) in the high mean treatment group, which was 77% ([58%, 92%]) and 80% ([66%, 93%]) of the total effect of the three mechanisms ($\hat{T}$), respectively. The main effect of nonlinear averaging in the low mean treatment was stronger but indistinguishable from zero (RGR: 0.052 SD [−0.053 SD, 0.14 SD]; herbivory: 36% [−20%, 130%]). Herbivore food selectivity in the high mean dose treatment group also increased caterpillar RGR and herbivory through a reduction in the mean toxin dose ingested (S) and an interaction between nonlinear averaging and food selectivity ($J_{S}$). The main effect of food selectivity increased caterpillar RGR by 2.2 × 10^−3^ SD ([3.8 × 10^−4^ SD, 4.9 × 10^−3^ SD]) and odds of herbivory by 1.9% ([0.44%, 4.0%]), which was 22% ([7.2%, 42%]) and 19% ([6.4%, 33%]) of the total effect of the three mechanisms ($\hat{T}$), respectively. Additional effects of food selectivity through nonlinear averaging were even smaller, increasing caterpillar RGR by only 6.1 × 10^−5^ SD ([8.5 × 10^−6^ SD, 1.4 × 10^−4^ SD]) and the odds of herbivory by 0.066% ([0.019%, 0.12%]) over 24 h. This positive interaction between food selectivity and nonlinear averaging in the high mean dose treatment group occurred because food selectivity shifted the dietary toxin distribution to a more convex region of the dose response functions (Appendix S1: Figure S3a,b), not because the variance of the dietary toxin distribution increased (Appendix S1: Section S3, Appendix S1: Figure S3).

## DISCUSSION

We tested the long‐standing hypothesis that quantitative variation in the level of plant defense between and within individuals may enhance the fitness of plants facing herbivory (Herrera, 2009; Karban et al., 1997; Pearse et al., 2018; Schultz, 1983; Whitham, 1981). Our results provide partial support for this hypothesis. Plants can indeed acquire greater fitness with just a moderate increase in the level of defense variation. A similar reduction in herbivory with greater variation in glucosinolate has been reported in *Brassica oleracea* at the inter‐individual scale (Bustos‐Segura et al., 2017) and may explain the common observation of neighborhood‐level phytochemical overdispersion in nature (Wang et al., 2023), potentially driving important processes such as plant–plant coexistence (Salazar & Marquis, 2022). At the intraindividual scale, variable allocation of toxin may itself be a form of plant defense, akin to traditional optimal defense theory that saw wide success in explaining patterns of mean glucosinolate allocation (Tsunoda et al., 2017). Given recent work indicating that variation might be heritable (Ayroles et al., 2015; Herrera et al., 2022; Hill & Mulder, 2010; Jimenez‐Gomez et al., 2011), selection for intraindividual trait variation is possible (Bruijning et al., 2020; Herrera, 2009; Metcalf & Ayroles, 2020), raising the intriguing hypothesis that widespread shared selective pressure may explain the maintenance of the tremendous variation in plant defense traits frequently observed in nature (Wetzel et al., 2023), including for *A. thaliana* (Katz et al., 2021; Panthee et al., 2011). Importantly, our result suggests that these variations have an ecological function beyond simple metabolic cost saving (Züst et al., 2011). Where the capability to produce defense is limited, variability can allow a small amount of defense to go a long way. Given that processes outlined in Figure 1A–E are quite general, these conclusions are likely to extend beyond our model system to other herbivores, plants, and toxins.

However, our study shows that the benefit of defense variation is only present when plants are not well defended. When the plants are better defended, variation reduces plant fitness and benefits herbivore performance. Glucosinolate concentration in wild‐type *A. thaliana* naturally ranges from 0.25 to 4.25 nmol/mg among leaves (Hunziker et al., 2021), with many accessions exhibiting much higher concentrations. A reversal in the effect of variation was observed, with only a moderate (1.23 nmol/mg) increase in mean concentration from the wild‐type endogenous mean in our study. This reversal is because variation creates weak points in plant defense that are susceptible to selective attack from the herbivores. But more importantly, highly defended tissues have diminishing returns on their per unit defensive value (Figure 1D). This result may lead to the common observation that constitutively weakly defended plants are more inducible than constitutively highly defended plants (Kempel et al., 2011). Indeed, variation or additional defenses that arise from induced responses are only beneficial to the plant at low levels of defense, but not as useful at high levels of defense. Interestingly, many stochastic processes for positive finite random variables (e.g., plant defenses) generate a positive mean–variance relationship, where higher mean begets higher variance (Cohen & Xu, 2015; Giometto et al., 2015). Thus, the positive mean–variance relationship tends to oppose selective pressure.

That the effect on plant fitness and herbivore resistance is reversed at high defense levels agrees with existing theoretical models that include herbivore food selectivity. Thiel et al. (2021) showed that herbivore fitness should increase with defense variability when there is sufficiently strong food selectivity, especially for generalists. Shelton (2004) predicted that caterpillars can escape high concentrations of glucosinolates when the cost of moving is low. Indeed, we found that when plants have a high chemical defense concentration, stronger foraging preference allowed herbivores to circumvent plant defenses when the level of defense is variable. While the effect was trivially weak in our study, a result of how flat the dose–response function was across the doses we examined, the effect may take on greater importance when the herbivore is less tolerant or is more avoidant of the toxin; *T. ni*, as a diet generalist, occupies the extreme end of being not particularly choosy and sensitive to glucosinolates. Rather, our results suggest that a weakening of the effect of failure to physiologically track variation was a better explanation for the observed reversal. Although little is known about the dynamics of herbivore detoxification ability, promising work on modeling thermal tolerance suggests that the effects of variation in toxin experience may be predicted with relatively simple models (Rezende et al., 2020).

Our results underscore the importance of considering the mean and variation in concert (Benedetti‐Cecchi, 2003; Inouye, 2005), without which one may generate incorrect predictions about the effects of variability. Indeed, because studies of variability generally isolate the role of variability by holding mean conditions constant at a single level, they may overlook mean–variance relationships and mean–variance interactions that are pervasive in nature (Benedetti‐Cecchi, 2000; Taylor, 1961). In the few studies that have manipulated mean and variance independently, workers have often found completely opposite effects of variance depending on the mean. For instance, thermal variance is known to enhance insect performance at low temperatures but suppress it at high temperatures (Bozinovic et al., 2011; Kingsolver et al., 2015).

This context dependency becomes relevant when plant–herbivore researchers pick plant–herbivore species combinations that allow for herbivores' persistence so that data can be collected. However, herbivores interact frequently with non‐hosts in nature, where plant defenses are likely to be most relevant for protection against herbivory. For example, milkweed (*Asclepias*) defenses are typically studied using milkweed specialist herbivores, even though milkweed defenses are most effective against the many potential herbivores in their regional pool that are not specialists. We argue that this methodological bias might contribute to the prevailing view that plant variability contributes to herbivore suppression (Karban, 2011; Pearse et al., 2018; Shelton, 2004), when variability can have opposite effects on herbivores feeding on marginal hosts, possibly even allowing these herbivores to expand their realized niche. Just as a few high‐quality host plant individuals can drive the regional population dynamics of herbivores (Chesson, 2012; Helms & Hunter, 2005), variability could benefit individual herbivores by allowing them to exploit areas of low defense.

## CONCLUSION

We showed that defense variability has contrasting effects on plant fitness and herbivore resistance depending on the overall level of plant toxicity. These effects were likely caused by a combination of opposing mechanisms. Counterfactual simulations such as the ones employed in this study represent a powerful approach to disentangle these mechanisms and generalize their effects under a single unified framework. Future studies may include predators (Hauri et al., 2021) and the physiological cost of producing variable toxins (Bekaert et al., 2012) explicitly omitted from this study. While the net effect of variability, as well as the relative contributions of each mechanism, would likely depend on the particular biological system, our results suggest that failure to physiologically track variation in defenses may be generally less detrimental at higher defense levels. Because the biological activity of all toxins depends on their accumulation or the accumulation of their damage in an herbivore's system through time, the proportional effects of variation on the time to critical toxin accumulation at high concentrations are much lower than at low concentrations.

Our study offers one explanation for the large variation in plant defense traits observed in nature, with implications for the practice of crop diversification (Isbell et al., 2017). While crop diversification has been proposed to enhance pest suppression by increasing plant trait diversity in agricultural fields (Pearse et al., 2018; Wetzel et al., 2016), our study suggests this practice might sometimes backfire for well‐defended plants and herbivore pests at the margins of their niche. This fact might explain the inconsistent effects of crop diversification and plant genetic diversity on herbivores reported in several meta‐analyses (Dassou & Tixier, 2016; Koricheva & Hayes, 2018; Tonhasca Jr & Byrne, 1994). Recently, Holmes and Blubaugh (2023) noted that evidence of plant diversity suppressing herbivore populations comes almost exclusively from agroecosystems, whereas evidence from natural systems is often mixed or positive. Considering humans artificially select for crop plants that express low levels of plant defenses, the observation appears to be consistent with our results. Thus, while trait diversity in agroecosystems often helps suppress pests, our results suggest a key limitation. Understanding and predicting the effects of functional variation would require considering mean–variance relationships and mean–variance interactions that are pervasive in nature.

## CONFLICT OF INTEREST STATEMENT

The authors declare no conflicts of interest.

## Supporting information


Appendix S1.

## Data Availability

Data (Pan et al., 2024a) are available in Dryad at https://doi.org/10.5061/dryad.cz8w9gjbm. Code (Pan et al., 2024b) is available in Zenodo at https://doi.org/10.5281/zenodo.10957003.
