# Supplementary material for: Mean plant toxicity modulates the effects of plant defense variability
Source: Ecology. 2025 Feb 4;106(2):e70012. doi: 10.1002/ecy.70012 (PMC11792111; doi:10.1002/ecy.70012)
Supplement: Supplementary file 1 — Appendix S1. [file ECY-106-e70012-s001.pdf]

Ecology

Mean plant toxicity modulates the effects of plant defense variability

Vincent S. Pan, Kadeem J. Gilbert, William C. Wetzel

Section S1: Dose calibration

We used sinigrin potassium salt ($C_{10}H_{16}KNO_9S_2$) or its impure form sinigrin potassium monohydrate ($C_{10}H_{18}KNO_{10}S_2$) as the manipulated plant defense trait in our experiments. Sinigrin, or Allyl glucosinolate, is a type of glucosinolate that *Arabidopsis thaliana* produces (Harun et al. 2020). Panthee et al. (2011) found that it consists of on average ~12% of the total glucosinolates produced across 58 *A. thaliana* ecotypes accessed. To determine the molar concentration of glucosinolate we deployed on *A. thaliana* leaves through painting, we first determined the amount of water that each milligram of *A. thaliana* leaf tissue holds. To do so, we selected 48 leaves from 10 plants and measured the weight of the leaf before and after it was painted with water. We subtracted the difference to get the amount of water that is coated on the leaf. We found that on average, a milligram fresh weight of leaf tissue can hold 0.32 mg (89% CI = [0.12, 0.55]) of water. We then use the molar mass of sinigrin potassium monohydrate or sinigrin potassium salt and the density of water to calculate the target salt mass per volume of water for a given dose (Table S1).

Section S2: Steps for variance decomposition

1. Fit generalized additive model to dose-response data to estimate the dose-response function $f(x)$ along a toxin concentration gradient x. Denote the ith posterior draw of a dose response function as $f^i(x)$.
2. Fit a model to estimate the relative preference for food with different concentrations of toxin x. Denote the jth posterior draw of the observed preference as $w_o^j(x)$. In our study, we used the number of leaves of each concentration class consumed as a measure of $w_o(x)$.
3. Define the probability density function of the observed distribution of dietary toxin dose experienced by an herbivore X_o^j by renormalizing preference weights for each concentration level $w_o^j(x)$ over all possible values of x, $P\left(X_o^j = x\right) = \frac{w_o^j(x)}{\int_0^\infty w_o^j(x)dx}$.
4. Define the probability density function of the counterfactual distribution of dietary toxin dose experienced by a randomly feeding herbivore X_r using the same method as above but with $w_r(x) \sim A(x)$, where $A(x)$ is the frequency at which a concentration x is available in the environment. In our study, there were two concentrations used, each with equal frequency of availability.
5. Compute the main effect of nonlinear averaging (without the effect of selective feeding) using its definition, $J_r^i \coloneqq \mathbb{E}\left[f^i(X_r)\right] - f^i(\mathbb{E}[X_r])$. Here, $\mathbb{E}\left[f^i(X_r)\right]$ is the average of a collection of nonlinear responses and $f^i(\mathbb{E}[X_r])$ is the average concentration mapped to a location on the nonlinear response function. $\mathbb{E}\left[f^i(X_r)\right]$ can be evaluated using the probability density computed earlier as $\int_0^\infty f^i(x)P(X_r = x)dx$.

6. Compute the effect of nonlinear averaging with the effect of selective feeding using its definition, $J_o^{i,j} \coloneqq \mathbb{E}\left[f^i\left(X_o^j\right)\right] - f^i\left(\mathbb{E}\left[X_o^j\right]\right)$.
7. Compute the marginal effect of the interaction between selective feeding and nonlinear averaging, $J_S^{i,j} = J_o^{i,j} - J_r^i$.
8. Compute the main effect of selective feeding, independent of nonlinear averaging, $S^{i,j} = f^i\left(\mathbb{E}\left[X_o^j\right]\right) - f^i(\mathbb{E}[X_r])$. For a sanity check, we can find that if selective feeding acts on the distribution of X only, the total effect of selective feeding (including its interactions) must be $\mathbb{E}\left[f^i\left(X_o^j\right)\right] - \mathbb{E}\left[f^i(X_r)\right] \coloneqq S^{i,j} + J_S^{i,j}$, which leads to $\mathbb{E}\left[f^i\left(X_o^j\right)\right] - \mathbb{E}\left[f^i(X_r)\right] = S^{i,j} + \left(\mathbb{E}\left[f^i\left(X_o^j\right)\right] - f^i\left(\mathbb{E}\left[X_o^j\right]\right)\right) - \left(\mathbb{E}\left[f^i(X_r)\right] - f^i(\mathbb{E}[X_r])\right)$, yielding the definition of $S^{i,j}$ again.
9. Compute the total predicted effect $\hat{T}^{i,j} \coloneqq J_r^i + S^{i,j} + J_S^{i,j}$.
10. Compute total missing effects $\epsilon^{i,j,k} \coloneqq T_o^k - \hat{T}^{i,j}$, where T_o^k is the kth posterior draw of the observed effect.
11. Repeat steps 1 – 10 for different values of i, j, k and summarize results. To facilitate comparison across variables, we standardized the estimated effects by the standard deviation of the response variable in the variation treatment experiment (Figure S1b).

Section S3: Causes of the positive interaction effect J_S

When we detected a positive interactive effect between herbivore food selectivity and nonlinear averaging, we conducted further simulations to investigate whether this was due to a change in the variance or the mean of the distribution of dietary toxin. Consider a second order Taylor approximation of the nonlinear averaging effect,

$$J \approx \frac{1}{2}\frac{\partial^2}{\partial x^2}f(\mathbb{E}[X])\mathbb{V}[X], \tag{S1}$$

where $\frac{\partial^2}{\partial x^2}f(\mathbb{E}[X])$ denotes the degree of convexity at the mean dietary toxin level $\mathbb{E}[X]$ and $\mathbb{V}[\cdot]$ denotes the variance. Food selectivity could have shifted the local convexity to a more positive region or increased the variance in dietary toxin, resulting in a stronger positive nonlinear averaging effect. To distinguish these possibilities, we compared the mean and variance of dietary toxin with or without food selectivity in our simulations. We also compared the degree of local convexity, approximated via second order forward finite difference.

Our simulations revealed that the source of the positive interaction J_S in the high mean dose treatment group between food selectivity and nonlinear averaging arose from a reduction in mean dietary toxin in the presence of food selectivity (-0.037 [-0.068, -0.011] nmol / mg, Figure S3a, eqn. S1). This shift in mean dietary toxin distribution corresponded to a more convex region of the RGR and herbivory dose response function (RGR: 1.6×10^{-5} [2.0×10^{-6}, 3.9×10^{-5}] hour^{-1} / (nmol/mg)^2; herbivory: 0.015 [0.0034, 0.031] logit / (nmol/mg)^2; Figure S3b). In fact, this increase in convexity more than compensated for the reduction in variance in dietary toxin (-0.30 [-0.46, -0.14] (nmol/mg)^2, Figure S3a). Conversely, food selectivity did not modulate nonlinear averaging in the low cage mean dose treatment groups because the change in dietary toxin variance was not large enough and there was no change in mean dietary toxin (Figure S3a).

References

Harun, S., Abdullah-Zawawi, M.-R., Goh, H.-H. & Mohamed-Hussein, Z.-A. (2020). A Comprehensive Gene Inventory for Glucosinolate Biosynthetic Pathway in *Arabidopsis thaliana*. *J. Agric. Food Chem.*, 68, 7281–7297.

Panthee, D.R., Kopsell, D.A. & Sams, C.E. (2011). Diversity Analysis of *Arabidopsis thaliana* (L.) Heynh Ecotypes for Glucosinolates in Shoots and Seeds. *HortScience*, 46, 968–974.

Table S1. The amount of glucosinolate in different cage mean by variation treatment combinations. The variation treatment (either intra- or inter-individual) varied between a ‘low’ or a ‘high’ within cage treatment. The constant treatment kept the concentration at the ‘mean’ dose. The dose of glucosinolate (sinigrin potassium) is expressed in nmol per mg of *A. thaliana* fresh weight. The sinigrin potassium monohydrate and sinigrin potassium salt concentration are in mg of dry powder per 1 ml of water.

Cage mean treatment	Variation treatment	Dose (nmol / mg)	Sinigrin potassium monohydrate concentration (mg / ml)	Sinigrin potassium salt concentration (mg / ml)
Low	Low	0.051	0.066	0.063
Low	Mean	0.66	0.86	0.82
Low	High	1.27	1.65	1.58
High	Low	1.27	1.65	1.58
High	Mean	1.89	2.45	2.34
High	High	2.50	3.24	3.1

Table S2. Sources of key materials used in the experiment. The soil used in all experiments is a mix of 50/50% SureMix and LM-GPS.

**Material**	**Source**
SureMix	Michigan Grower Products, Inc., Galesburg, Michigan, USA
LM-GPS	Lambert Peat Moss, Rivière-Ouelle, Québec, Canada
Caterpillars (*Trichoplusia ni*)	Benzon Research, Carlisle, Pennsylvania, USA
Col-1 *Arabidopsis thaliana*	Nicole Parker, Oklahoma State University, Department of Plant Biology, Ecology, and Evolution
Sinigrin potassium salt	Sigma-Aldrich, Inc., St. Louis, Missouri, USA
Sinigrin hydrate	Sigma-Aldrich, Inc., St. Louis, Missouri, USA

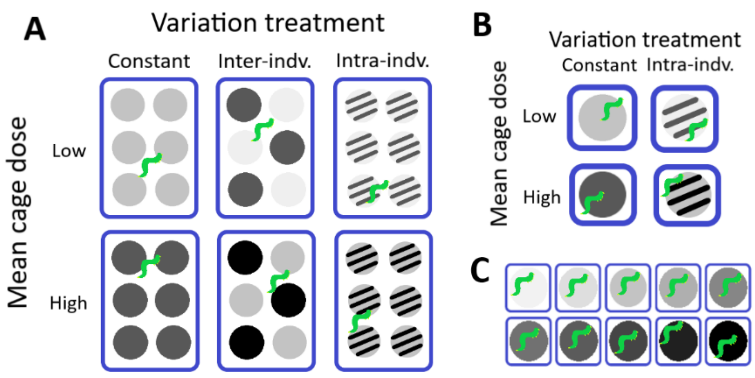

Figure S1. Design of (a) field experiment for assessing plant fitness, (b) greenhouse experiment for assessing herbivore performance and herbivory, and (c) dose-response experiment for parameterizing simulation. The blue boxes represent individual cages containing one caterpillar each. Each circle represents one plant, with the level of the shading representing the painted dose of the sinigrin (darker shading is higher concentration). Circles with stripes represent plants with the intra-individual variation treatment (different sinigrin concentrations painted onto alternate leaves).

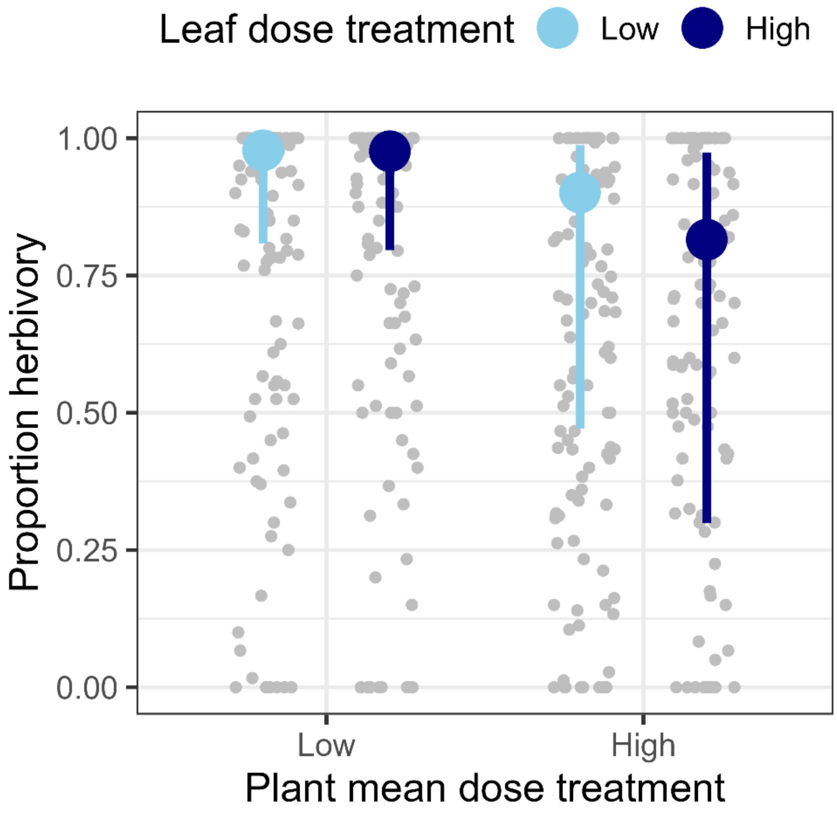

Figure S2. Proportion of herbivory on leaves painted with a low or high sinigrin dose in different mean plant level dose treatment groups. The difference in herbivory among leaf dose treatments is significant and greater in the treatments with a high mean plant dose. Colored points represent mean estimates. Colored bars represent 89% credible intervals. Each grey point represents a leaf.

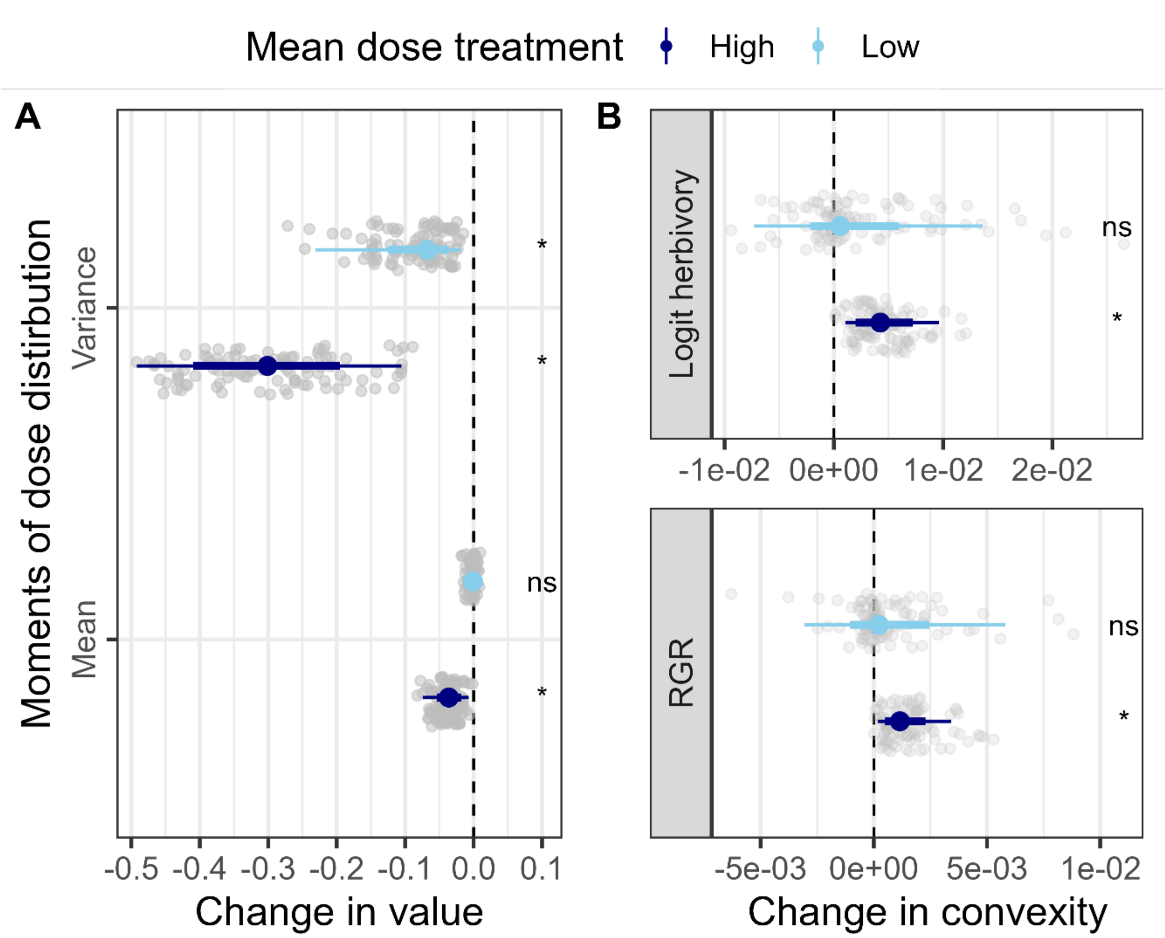

Figure S3. A second order Taylor approximation of nonlinear averaging (eqn. S1) suggests that a positive interactive effect between nonlinear averaging and selective feeding on herbivory and relative growth rate (RGR) must arise from an increase in the variance $\mathbb{V}[X]$ or the local convexity of the dose response function $\frac{\partial^2}{\partial x^2} f(\mathbb{E}[X])$. Simulation revealed that the interactive effect arose from a shift in the mean dose distribution $\mathbb{E}[X]$ experienced by the herbivore, which maps to a more convex region of the dose-response function $f(\mathbb{E}[X])$. (a) Change in mean (nmol/mg) or variance (nmol^2/mg^2) of the distribution of dietary toxin due to herbivore food selectivity under different meant treatment conditions. (b) Change in standardized local convexity (second derivative) of the dose-response function in response to shifts in mean dietary toxin due to herbivore food selectivity (standard deviation / (nmol/mg)^2). Colored points, thin

bars, and thick bars represent 50%, 66%, and 89% CIs respectively. A random sample of 100 simulations are plotted as grey points. An asterisk indicates if the 89% CIs do not overlap zero (black dashed line), and ‘ns’ denotes a nonsignificant effect.
